# Caprine *PRNP* polymorphisms N146S and Q222K are associated with proteolytic cleavage of PrP^C^

**DOI:** 10.1186/s12711-021-00646-x

**Published:** 2021-06-19

**Authors:** Sally A. Madsen-Bouterse, Paula Stewart, Helen Williamson, David A. Schneider, Wilfred Goldmann

**Affiliations:** 1 Department of Veterinary Microbiology and Pathology, College of Veterinary Medicine, Washington State University, Pullman, WA USA; 2 The Roslin Institute and R(D)SVS University of Edinburgh, Easter Bush, Midlothian, UK; 3 Animal Disease Research Unit, Agricultural Research Service, US Department of Agriculture, Pullman, WA USA

## Abstract

Expression of the cellular prion protein (PrP^C^) is crucial for the development of prion diseases. Amino acid changes in PrP^C^ or a reduced amount of PrP^C^ may modulate disease resistance. The relative abundance of C1, a natural α-cleavage fragment of PrP^C^, was previously found to be associated with a resistant *PRNP* genotype in sheep. Goats are another small ruminant where classical scrapie susceptibility is under strong genetic control. In this study, we assessed PrP^C^ in goats for the existence of similar associations between PrP^C^ fragments and genotype. Brain tissue homogenates from scrapie-free goats with wild type *PRNP* or polymorphisms (I142M, H143R, N146S, or Q222K) were deglycosylated prior to immunoblot for assessment of the relative abundance of the C1 fragment of PrP^C^. The presence of K_222_ or S_146_ alleles demonstrated significantly different relative levels of C1 compared to that observed in wild type goats, which suggests that the genotype association with C1 is neither unique to sheep nor exclusive to the ovine Q171R dimorphism.

**Supplementary Information:**

The online version contains supplementary material available at 10.1186/s12711-021-00646-x.

## Background

Expression of the cellular form of the prion protein (PrP^C^) is essential for the pathogenesis of a group of disorders called prion diseases, also known as transmissible spongiform encephalopathies (TSE). Prion diseases include Creutzfeldt–Jakob disease (CJD) in man, chronic wasting disease (CWD) in deer, bovine spongiform encephalopathy (BSE) in cattle and scrapie in sheep and goats. Prion diseases are marked by the accumulation of protease-resistant isoforms of the prion protein, designated PrP^Sc^, in the central nervous system [1]. Accumulation of PrP^Sc^ occurs by a mechanism of seeded conversion of PrP^C^ which can result in PrP^Sc^ aggregates ranging from discrete fibrils to diffuse amyloid plaques [2].

PrP^C^ is encoded by the *PRNP* gene and is most highly expressed in the central nervous system [3, 4]. The biological function of PrP^C^ is not clear, but roles in cell survival, circadian rhythm, myelin maintenance, and immunity have been suggested (reviewed by [5]). PrP^C^ from brain tissue is readily visualized by immunoblot using prion protein-specific antibodies. Before enzymatic deglycosylation, PrP^C^ occurs in three bands with apparent molecular weights of 33–35 kDa (di-glycosylated), 30–32 kDa (mono-glycosylated) and 25–27 kDa (un-glycosylated), all bearing a GPI-anchor. The PrP^C^ bands detected after deglycosylation typically have apparent molecular weights of 26 kDa, 18 kDa and 16 kDa, representing full-length PrP^C^ (209 amino acids) and two shorter fragments generated by proteolytic cleavage ([6] and reviewed in [7, 8]). Of the two shorter fragments, the 16 kDa band represents a 120 amino acid C-terminal fragment (C1) produced by the so-called α-cleavage between histidine_114_ and valine_115_. The 18 kDa band represents an approximately 142 amino acid C-terminal fragment (C2) produced by β-cleavage at or near glycine_92_. The C2 fragment tends to occur at low levels (relative to C1) in healthy animals and may represent a response to oxidative stress [8].

It is a long held view that incubation periods of prion diseases are correlated with the amount of PrP^C^ available for conversion to PrP^Sc^. This has been demonstrated in various transgenic mouse models in which incubation periods of experimentally-induced prion disease are inversely correlated with the expression level of mature full-length PrP^C^ [9, 10]. However, the C1 fragment does not convert into a protease-resistant isoform in scrapie-challenged transgenic mice expressing only C1 [11]. Furthermore, co-expression of C1 with full-length PrP^C^ resulted in extended incubation periods. Likewise, cell lines with higher levels of C1 are relatively less permissive to prion infection [12]. This raises the possibility that PrP^C^ cleavage may modulate prion diseases by reducing the amount of full-length PrP^C^ available for conversion or C1 fragment-mediated inhibition of full-length PrP^C^ conversion. Interestingly, a laboratory-generated PrP^C^ deletion mutant was found to undergo spontaneous conversion to a prion of similar size as the C1 fragment [13]. This new type of prion was also able to infect and propagate in a cell line that expressed the deletion-mutant PrP^C^ C1 fragment only. While these observations suggest that the C1 fragment may be susceptible to prion conversion in the presence of additional modifications, the C1 fragment of natural PrP^C^ appears to be relatively resistant to both spontaneous and seeded prion conversion.

Polymorphisms in the *PRNP* gene that encode PrP^C^ protein sequence variants (allotypes) can impact prion disease susceptibility and incubation period (reviewed in [14, 15]). In sheep, the proteolytic processing of PrP^C^ in the brain depends on the *PRNP* genotype [16], such that the relative amount of the C1 fragment is increased and that of the C2 fragment is decreased in *PRNP* genotypes associated with resistance to classical scrapie. Similar co-associations of PrP^C^ allotypes with proteolytic processing and disease phenotypes remain to be investigated in other species. In this study, the relative amounts of PrP^C^ and C-terminal fragments present in the brain of scrapie-free goats were compared for PrP^C^ allotypes known to be either associated with delayed incubation or reduced susceptibility risk of classical scrapie.

## Methods

### Study population

All animals were maintained under animal care using protocols approved by the Washington State University Institutional Animal Care and Use Committee, or the University of Edinburgh Animal Welfare and Ethical Review Body. In an effort to include as genetically diverse a population as possible, 50 goats of various breeds from two geographically distinct countries were used (ADRU/WSU n = 25 and Roslin n = 25). All goats were confirmed as scrapie negative or ‘not detected’ in the post-mortem examination of formalin-fixed lymph node and brain tissues by standard laboratory diagnostic methods [17, 18]. *PRNP g*enotypes were determined by DNA sequence analyses as previously described [19, 20] and confirmed according to protocols described by Goldmann and colleagues [21, 22]. More than 50 *PRNP* gene polymorphisms have been identified in goats, many of which encode PrP^C^ allotypes [20, 23]. Polymorphisms that encode a serine (S) or a proline (P) at codon 240 are both considered to be wild type for caprine *PRNP* and are susceptible to scrapie [24, 25]*.* Additional polymorphisms appear in combination with S240P and some of these are associated with modulated susceptibility, incubation periods, or pathology of prion diseases (reviewed by [14]). Of interest in the current study are goat genotypes defined by polymorphisms that result in amino acid changes at codons 142 (isoleucine to methionine, I142M), 143 (histidine to arginine, H143R), 146 (asparagine to serine, N146S), and 222 (glutamine to lysine, Q222K). Since the amino acid encoded by codon 240 is not present in mature full-length PrP^C^, the relative fragmentation of PrP^C^ was determined for the available allele combinations where I_142_H_143_N_146_Q_222_ is wild type.

### Brain tissue preparations

All tissues were stored at − 70 °C and transported at − 20 °C. Each sample of brain tissue (cortex, brainstem and (or) cerebellum) was manually homogenised in lysis buffer (5% NP-40 (v/v), 12.1 mM sodium deoxycholate in PBS) with protease inhibitors (10 µM PMSF, 10 µM NEM or Complete Mini Tablets, Roche) to make a 10% (w/v) homogenate. The homogenate was clarified by centrifugation at 400*g* at 4 °C for 10 min, the supernatant was collected, flash frozen and stored at − 20 °C until further analysis.

### Deglycosylation

Brain homogenate (10% w/v) was denatured at 100 °C for 10 min and incubated with 0.125U of peptide *N*-glycoside F (PNGaseF kit, New England Biolabs) at 37 °C for 2 h according to manufacturer’s instructions. Deglycosylated protein was precipitated with methanol and stored at − 20 °C. Before immunoblotting, protein was pelleted by centrifugation at 10,400*g* for 10 min at 4 °C. Samples were suspended in NuPAGE sample buffer (Invitrogen) supplemented with a reducing agent (Invitrogen).

### SDS-PAGE and immunoblotting

Deglycosylated protein was denatured at 70 °C for 10 min and separated on 12% NuPAGE Bis–Tris gels (Invitrogen) or 12% Criterion gels (BioRad). Molecular markers spanning 10–250 kDa were used for size reference (MagicMarker XP Western protein standard, Invitrogen; Precision Plus Protein Western C standard, Bio-Rad) and electrophoresis was performed in an Xcell SureLock tank at 150 V for 1 h using a NuPAGE™ MOPS SDS buffer kit for Bis–Tris gels (Invitrogen) or 20× XT MOPS running buffer kit (BioRad). Proteins were transferred onto polyvinylidene difluoride membranes (PVDF; Millipore or GE Healthcare) at 25 V for 1 h, after which the membranes were washed with TBS (50 mM Tris, 150 mM NaCl, pH 7.5). The membranes were blocked using 1% (v/v) blocking solution in TBS (Western Blocking Reagent; Roche) for 1 h at room temperature under agitation followed by incubation with PrP-specific antibodies and anti-tubulin (protein loading control, anti-murine α-tubulin IgG_1_ 0.01 μg/mL; Fisher Scientific) diluted in 0.5% (v/v) blocking solution under the same conditions. Prion protein specific monoclonal antibodies included BC6 (0.1 μg/mL), FH10 (0.5 μg/mL), F89/160.1.5 (3.5 μg/mL) and F99/97.6.1 (3.5 μg/mL) (Table 1). Antibodies BC6 and FH10 were obtained from the TSE Resource Centre, The Roslin Institute, University of Edinburgh [26]; antibodies F89.160.1.5 and F99/97.6.1 were prepared from hybridomas maintained at the USDA Animal Disease Research Unit [27, 28]. Membranes were washed with TBST (0.1% Tween 20 in TBS) followed by 0.5% (v/v) blocking solution. The membranes were incubated in horseradish-peroxidase-conjugated rabbit anti-mouse at 0.08 μg/mL (Stratech, UK) in 0.5% (v/v) blocking solution for 75 min. The membranes were washed in TBST and proteins were visualized using activated chemiluminescence (SuperSignal West Dura Extended Duration Substrate, Thermo Scientific) and Lumi-Film Chemiluminescent Detection Film (Roche).

**Table 1:** PrP-specific monoclonal antibodies used to detect full-length PrP^C^, C1, and C2

Antibody	Epitope^a^	Isotype	References^b^
BC6	^144^F**GN**DYED**R**YY**R**^154^	IgG_1_	[26]
FH10	^202^TETDIKIME^210^	IgG_2a_	[26]
F89/160.1.5	^142^**IH**F**G**^145^	IgG_1_	[28]
F99/97.6.1	^220^**Q**Y**Q**RES^224^	IgG_1_	[27]

^a^Amino acids in bold font may be altered due to single nucleotide polymorphisms known to occur in caprine *PRNP* [29]. Underlined amino acids are the focus of the current study

^b^First description of prion protein-specific antibody

### Data analysis

For quantitative analysis, blots were scanned and the net intensity of manually-selected protein bands that were representative of full-length PrP^C^, C1 and C2 were measured using Adobe Photoshop and (or) ImageJ. The relative amount of each band was expressed as a percentage of the sum of all three bands per animal. The median percentage of C1 was calculated for each goat from all observations detected with a single antibody (deglycosylation, immunoblot, and densitometry; the number of values contributing to the median for each antibody ranged from one to six per goat). The mean percentage of C1 was calculated from antibody medians for each goat. Allotype-associated differences in mean percentage of C1 were assessed using beta regression (PROC GLIMMIX; SAS version 9.4, SAS Institute Inc., Cary, NC, USA) with stepdown Dunnett post-hoc testing of comparisons relative to wild type. Significance was attributed to effects with *p*-values < 0.05.

## Results and discussion

### Relative abundance of PrP^C^ C1 fragment in goats with wild type *PRNP* and genotypes associated with delayed scrapie incubation

**Fig. 1:**
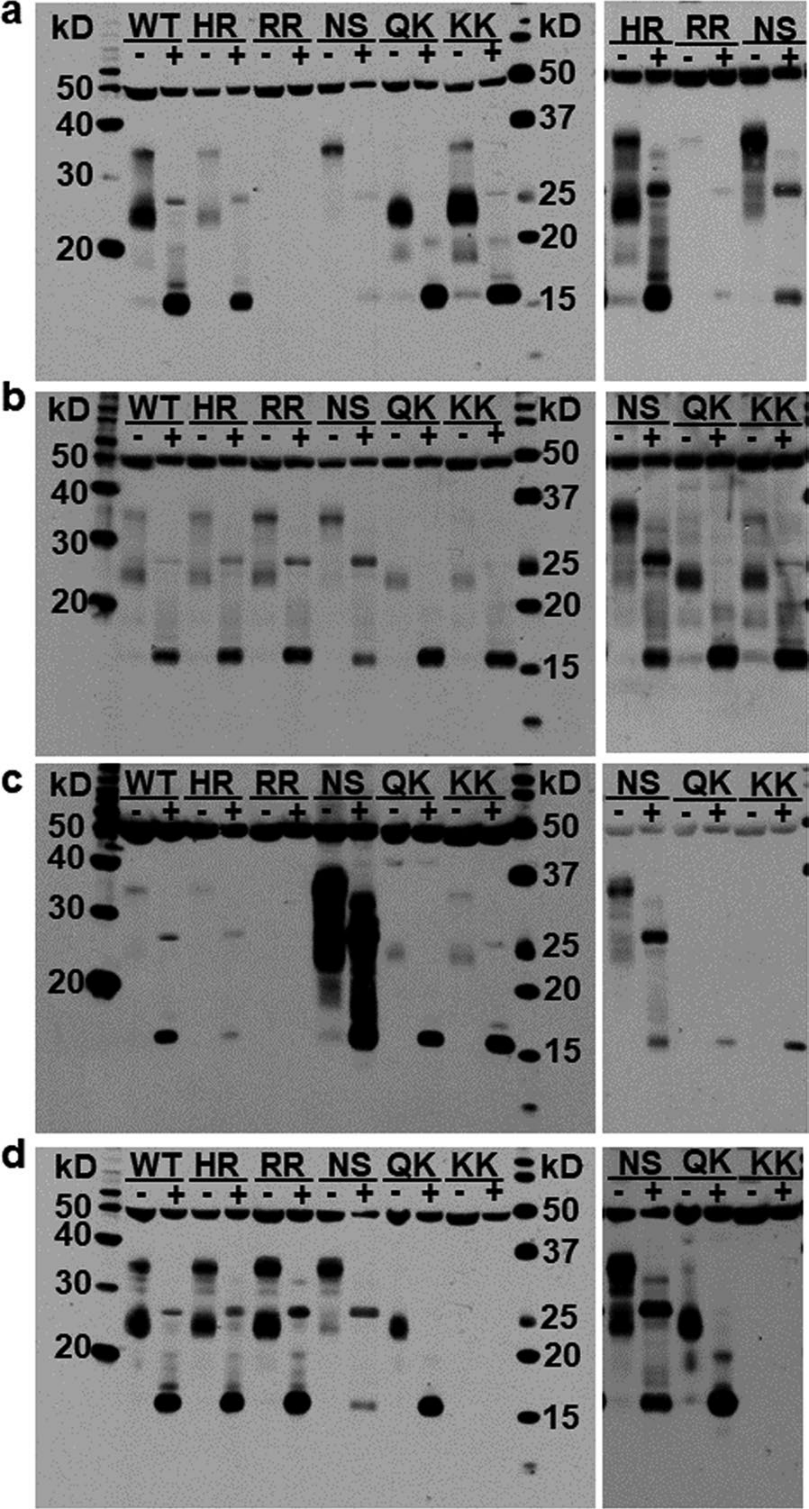
Goat PrP^C^ before and after deglycosylation. Representative immunoblots of cortex homogenate (20 µg total protein) prepared from goats of different genotypes before (−) and after (+) treatment with PNGaseF. Detection of tubulin (~ 50 kDa) was used to assess sample loading. In deglycosylated samples (+), full-length PrP^C^ is visible at ~ 26 kDa and the C1 fragment at ~ 16 kDa. Detection with anti-PrP antibodies BC6 (**a**), FH10 (**b**), F89/160.1.5 (**c**), and F99/97.6.1 (**d**) demonstrates the impact of alternative alleles on PrP^C^ detection. Additional panels show reduced detection by BC6 in RR_143_ goat (**a**), a longer film exposure to demonstrate the faint full-length PrP^C^ bands in samples with an allele encoding K_222_ (**b**), a shorter film exposure for NS_146_ goat (**c**), and panel in (**d**) shows lack of detection by F99/97.6.1 in KK_222_ goats even after a long film exposure. WT = II_142_–HH_143_–NN_146_–QQ_222_; HR = II_142_–HR_143_–NN_146_–QQ_222_; RR = II_142_–RR_143_–NN_146_–QQ_222_; NS = II_142_–HH_143_–NS_146_–QQ_222_; QK = II_142_–HH_143_–NN_146_–QK_222_; KK = II_142_–HH_143_–NN_146_–KK_222_

**Fig. 2:**
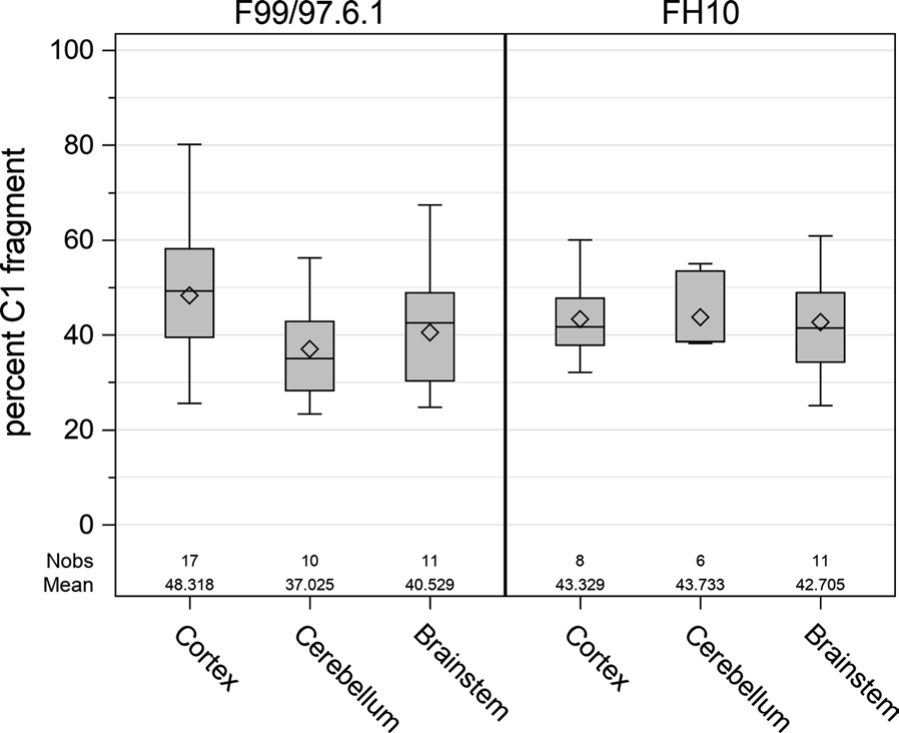
The region of the brain sampled does not significantly alter the relative abundance of C1 in goats. Cortex, cerebellum and (or) brainstem from a subset of goats were assessed by immunoblot using two different prion protein-specific antibodies. Brain region associated differences in percentage of C1 were assessed using beta regression (PROC GLIMMIX; SAS version 9.4, SAS Institute Inc., Cary, NC, USA). There were no significant differences due to the brain region sampled, the antibody used for detection (e.g. F99/97.6.1 versus FH10), or the interaction between brain region and antibody (*p* > 0.05)

The brain is the penultimate site of PrP^C^ conversion to PrP^Sc^ prior to the development of clinical signs associated with classical scrapie. Brain from each goat was collected at cull and frozen until homogenization, deglycosylation, and immunoblotting. The ability to detect PrP^C^ by immunoblot can depend on factors that affect antibody affinity and specificity. Previous studies have demonstrated that *PRNP* polymorphisms encoding amino acid changes can impact antibody-based prion protein detection [26, 30]. Thus, detection in this study was performed with several PrP-specific monoclonal antibodies (BC6, FH10, F89/160.1.5, or F99/97.6.1; Table 1) and detection of alpha-tubulin was used as a loading control (Fig. 1). All the PrP-specific antibodies bind epitopes that are present in full-length PrP^C^, C1, and C2 fragments in wild type goats (II_142_–HH_143_–NN_146_–QQ_222_). Most samples were tested with BC6, which was previously used in sheep [16]. The relative amount of C2 was very low in all goats and will not be discussed further except to note a similarly low amount of C2 in sheep with a *PRNP* genotype that is associated with resistance to classical scrapie [16]. For all goats, the relative abundance of C1 was determined in homogenates prepared from cortex. Brainstem and (or) cerebellum were also sampled in a subset of goats. The relative amount of C1 fragment to total PrP^C^ was consistent across the three brain regions (Fig. 2); thus, all observations from a single goat were combined regardless of the brain region sampled. Uniformity across brain regions is comparable to what was observed in sheep [16]. In the brain of wild type goats (n = 22), the mean (± standard deviation) percentage of C1 relative to total PrP^C^ was 46.9 ± 9.3% (Table 2). There were no significant differences between measurements based on BC6 alone (49.2 ± 11.9%, n = 22), FH10 alone (44.3 ± 9.2%, n = 18) or F99/97.6.1 alone (50.5 ± 10.2%, n = 9) (see Additional file 1:Table S1). Antibody F89/160.1.5 was only used for five of the 22 wild type samples, but the results were not significantly different from the other antibodies. Thus, the choice of antibody did not appear to influence the results for wild type goats.

Prion protein allotypes resulting from single *PRNP* polymorphisms had a significant effect on the relative amount of C1 (*p* = 0.002). Post-hoc testing using a stepdown Dunnett’s approach was used to evaluate which allotypes were significantly different from the wild type. Several goats included in this study had prion protein allotypes that have been associated with delayed incubation of scrapie. Three of the goats were of the IM_142_–HH_143_–NN_146_–QQ_222_ allotype and had mean C1 levels of 41.9 ± 10.4%, which was not significantly different from that of the wild type (Table 2). When the H143R allotype was considered (II_142_–HR/RR_143_–NN_146_–QQ_222_), the mean C1 level in heterozygous goats (HR_143_; n = 5) was 53.8 ± 6.7% whereas homozygous goats with the alternative allotype (RR_143_; n = 5) had a mean C1 of 47.2 ± 9.2%. These values were also not significantly different from that of the wild type. Thus, the relative amount of C1 in goats does not appear to be associated with amino acid changes at codons 142 and 143, which are *PRNP* polymorphisms linked to delayed incubation of classical scrapie in goats [25, 31].

**Table 2:** C1 percentage of total PrP^C^ by *PRNP* genotype in goats

*PRNP* Genotype 142–143–146–222	Number of goats	Mean C1% of total PrP^C^ (± standard deviation)
All goats	50	49.7 ± 13.5
Genotype		
II–HH–NN–QQ^a^	22	46.9 ± 9.3
IM–HH–NN–QQ	3	41.9 ± 10.4
II–HR–NN–QQ	5	53.8 ± 6.7
II–RR–NN–QQ	5	47.2 ± 9.2
II–HH–NS–QQ	5	33.7 ± 3.2*
II–HH–NS–QK	2	62.5 ± 32.3
II–HH–NN–QK	6	67.8 ± 11.8*
II–HH–NN–KK	2	60.9 ± 16.1

^a^II_142_–HH_143_–NN_146_–QQ_222_ is wild type caprine *PRNP*

^*^Mean percent C1 of total PrP^C^ that is significantly different from wild type (n = 22) at *p* < 0.05

### Relative abundance of PrP^C^ C1 fragment in goats with PRNP genotypes associated with reduced-risk of scrapie

**Fig. 3:**
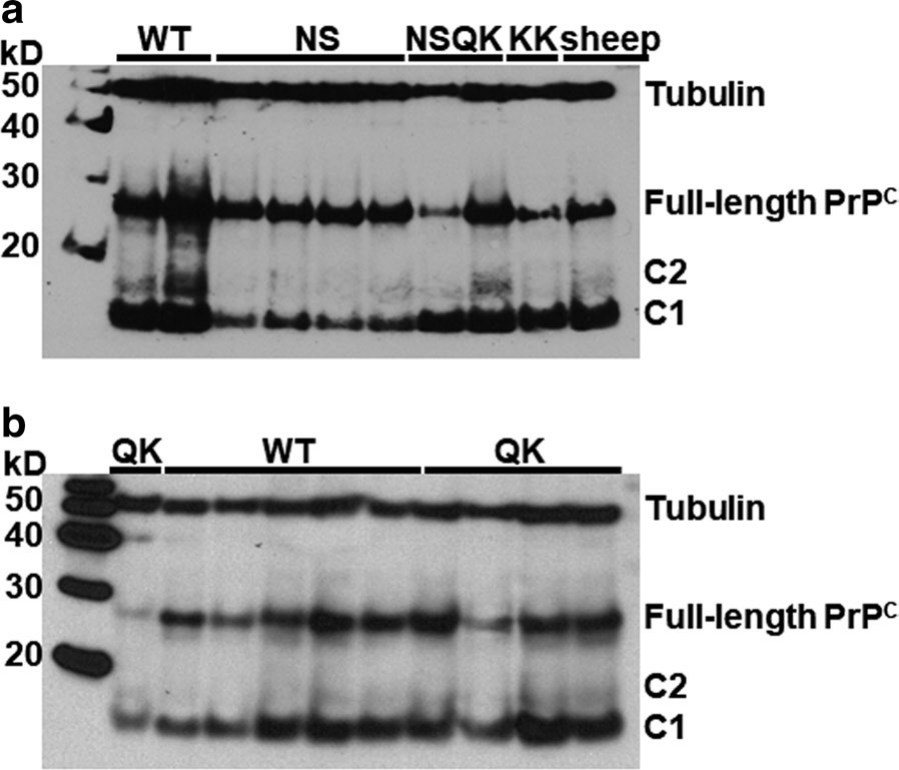
De-glycosylation of cellular prion protein reveals full-length PrP^C^ and fragments C1 and C2. Representative immunoblots of cortex homogenate (20 µg total protein) from goats with *PRNP* genotypes that are associated with resistance to classical scrapie. Following PNGaseF treatment, SDS-PAGE, and protein transfer to PVDF, immunodetection was performed with BC6 (**a**) and FH10 (**b**); detection of tubulin served as a protein loading control. WT = II_142_–HH_143_–NN_146_–QQ_222_; NS = II_142_–HH_143_–NS_146_–QQ_222_; NSQK = II_142_–HH_143_–NS_146_–QK_222_; QK = II_142_–HH_143_–NN_146_–QK_222_; KK = II_142_–HH_143_–NN_146_–KK_222_. Sheep (ARR/ARR) homogenate added for reference in panel (**a**)

A polymorphism encoding an amino acid change at codon 146 has been associated with a reduced risk and putative resistance to scrapie in goats [32–35]. The allele encoding S_146_ has been observed at a low frequency in meat goat breeds in Great Britain [21, 22] and some European Union member states [29]; S_146_ appears to be at a modestly higher frequency in meat breeds and some dairy breeds in the USA [20]. In this study, brain homogenates were available from five goats bearing the II_142_–HH_143_–NS_146_–QQ_222_ genotype. The mean C1 level in NS_146_ goats was 33.7 ± 3.2%, which was significantly lower than that of wild type goats (*p* = 0.0002 compared to wild type, n = 22) (Table 2 and Fig. 3a). The presumed epitope for antibody binding with BC6 [26] includes the amino acid at codon 146 (Table 1), thus, samples with NS_146_ were also tested with additional prion protein-specific antibodies. Differences between the percentage of C1 with BC6 versus with the other antibodies tested were not significant (see Additional file 1: Table S1). Hence, the observed decrease in percentage of C1 relative to that in wild type goats was not due to epitope-associated variation in PrP detection. Regardless of the antibody used for detection, there appeared to be more glycosylated PrP^C^ in brain homogenate from NS_146_ goats than from wild type (Fig. 1, samples that did not undergo treatment with PNGaseF prior to immunoblotting are indicated by a negative sign above the lane). All NS_146_ goats included in this study were of the Nubian breed whereas wild type goats were primarily Saanen or of mixed breed (mainly Saanen crosses), thus, the impact of genotype independently of the breed on PrP^C^ glycosylation could not be evaluated with the available samples. Whether PrP^C^ glycosylation has a direct impact on PrP^C^ proteolytic processing to yield the C1 fragment has yet to be investigated but observations in mice seem to suggest a role for glycosylation [36]. Although it was not the focus of the study by Wiseman and colleagues [36], the level of the C1 fragment was higher in glycosylation-deficient mice than in wild type mice or gene-targeted mice with reduced PrP^C^ glycosylation. The potential role of PrP^C^ glycosylation on the reduced abundance of the C1 fragment in NS_146_ goats is of interest and should be examined in future studies.

In recent years, the K_222_ allele has been of particular interest in goats because of the epidemiological evidence that suggests relative resistance of animals carrying this allele, and because of experimental studies that demonstrated a strong resistance to infection with scrapie in goats with this allele [22, 24, 29, 32, 33, 35, 37–40]. In the current study, 11 goats had at least one allele encoding K_222_. Goats with II_142_–HH_143_–NN_146_–QK_222_ had a significantly greater percentage of C1 (67.8 ± 11.8%, n = 6) relative to wild type (*p* = 0.02 Fig. 3b). The mean percentage of C1 in two goats homozygous for K_222_ (i.e., II_142_–HH_143_–NN_146_–KK_222_; 60.9 ± 16.1%) was similar to that of NN_146_–QK_222_ goats. Since we showed that the presence of the S_146_ allele in the NS_146_–QQ_222_ genotype reduced the mean level of C1 significantly, it might be expected that this was also the case in the two NS_146_–QK_222_ samples. However, the mean level of C1 observed in the NS_146_–QK_222_ samples was almost identical to the mean of the NN_146_–QK_222_ samples, but there was a large difference between the two NS_146_–QK_222_ samples (~ 45 points; see Fig. 3a and Additional file 1: Table S1). When we performed an immunoblot with F99/97.6.1 to measure only the PrP S_146_–Q_222_ allele [30, 41], the difference between both samples increased by more than 60 points, which is the largest difference observed at any single genotype. *PRNP* haplotypes in both goats were confirmed to be mutually exclusive (N_146_–K_222_ and S_146_–Q_222_) as was expected based on previous studies [20–22]. Additional animals with the NS_146_–QK_222_ allotype are needed to further understand the impact of this combination on the C1 fragment. Acquiring such samples may present substantial challenge because of the low prevalence of these individual alleles in the UK and USA goat populations [20–22] as well as populations from European Union member states [29].

We and others have previously demonstrated that F99/97.6.1 does not recognize the K_222_ allelic variant of PrP [30, 41, 42]. Thus, when F99/97.6.1 is applied to QK_222_ heterozygous samples in immunoblots, only the PrP-Q_222_ allotype (wild type/scrapie susceptible) is detected. Unexpectedly, in NN_146_–QK_222_ samples the mean for C1 from the single wild type PrP allele was 68.8 ± 13.4% (n = 6), which was 17.4 points higher than in homozygous wild type samples (50.5 ± 10.2%, n = 9) measured with F99/97.6.1 alone (see Additional file 1: Table S1). This suggests that the putative increased cleavage of the K_222_ variant is transmitted onto the wild type variant, which supports a dimerization model of PrP^C^ [43]. Together, these observations provide evidence for an allotype association with the percentage of C1 relative to full-length PrP^C^ in caprine brain.

### Relative abundance of PrP^C^ C1 fragment in goats versus sheep

In addition to the polymorphism at codon 240, the wild type genotype in goats shares an identical PrP^C^ sequence to the most common ovine allele that is described as ARQ in reference to alanine_136_, arginine_154_ and glutamine_171_. The average relative abundance of C1 in wild type goats (49.7%) is considerably higher than that in homozygous ARQ sheep (27.7%, [16]) and approaches the value reported for sheep that are homozygous for the scrapie resistant ARR genotype (52.6%, [16]). If the relative level of C1 is associated with incubation period length or susceptibility to scrapie, our data suggest that wild type goats may, in general, show longer incubation times and reduced susceptibility to scrapie than wild type sheep. Although this phenomenon has not been addressed directly by experimental challenge studies, we have observed longer incubation times in wild type goats versus ARQ sheep born to infected dams and raised in a similar manner in a persistently infected environment (see Additional file 2: Table S2 and Additional file 3: Figure S1). In addition, it appears that natural scrapie is less prevalent in goats than in sheep, unless they are in mixed holdings (reviewed in [29]). Furthermore, the relative amount of C2 was very low in all goats, mostly below the threshold of reliable measurement, which is another feature of caprine PrP^C^ processing that was similar to previous observations in scrapie-resistant ARR sheep [16].

## Conclusions

We have shown in a second species, i.e. goat, that an association exists between the relative levels of C1 and *PRNP* genotype. We measured the relative abundance of the C1 fragment as a percentage of total PrP^C^ in post-mortem brain from 50 healthy goats. Thirty-five goats were wild type (scrapie susceptible) or had genotypes that were associated with extended incubation of classical scrapie; 16 goats had putative scrapie-resistant genotypes (at least one S_146_ and(or) K_222_ allele present). Our aim was to investigate an association between the levels of the prion protein fragment C1 and *PRNP* genotype, as was previously shown in sheep [16]. Although goats of different breeds were included in this study, we could not perform an analysis independent of breed due to some genotypes being more prevalent in certain breeds [20, 21, 29]. Our observations show that resistant K_222_ expressing goats have significantly higher levels of C1 than wild type goats, which is similar to observations in scrapie susceptible Q_171_ expressing sheep versus R_171_ sheep that are resistant to classical scrapie. The increased percentage of C1 in goats with the K_222_ allele may have been an anticipated result based on observations in sheep but the significantly lower percentage of C1 in goats with the S_146_ allele was surprising considering that both *PRNP* genotypes are associated with classical scrapie resistance in goats. Previously, Eiden and colleagues [44] demonstrated a lack of PrP^Sc^-seeded conversion of PrP^C^ with the S_146_ and K_222_ genotypes. While the increased percentage of C1 in goats with the K_222_ allele may play a role in this phenomenon based on previous observations from prion infection studies using transgenic mice or prion permissive cell lines [11, 12], additional work is needed to understand how a lower percentage of C1 might contribute to the lack of seeded conversion in the presence of the S_146_ allele. Such experiments that could include an assessment of other factors, e.g. breed, are beyond the scope of this retrospective observational study. In summary, we propose that alterations in PrP^C^ processing may contribute to common mechanisms of differential susceptibility to protein misfolding and resistance to classical scrapie disease in goats as has been described in sheep.

## Supplementary Information

**Additional file 1: Table S1.** Mean percentage of the C1 fragment relative to total PrP^C^ for each animal and antibody.

**Additional file 2: Table S2.** Product-limit estimates of median survival (days) for naturally scrapie-infected sheep and goats homozygous for the wild type *PRNP* genotype including alanine_136_ and glutamine_171_.

**Additional file 3: Figure S1.** Graphical representation of survival curves for naturally scrapie-infected sheep and goats homozygous for the wild type *PRNP* genotype including alanine_136_ and glutamine_171_.

## Data Availability

The dataset supporting the conclusions of this article is included within the article (and its additional files).
