# Supplementary material for: Caprine PRNP polymorphisms N146S and Q222K are associated with proteolytic cleavage of PrPC
Source: Genet Sel Evol. 2021 Jun 19;53:52. doi: 10.1186/s12711-021-00646-x (PMC8214774; doi:10.1186/s12711-021-00646-x)
Supplement: Supplementary file 2 — Additional file 2: Table S2. Product-limit estimates of median survival (days) for naturally scrapie-infected sheep and goats homozygous for the wild type PRNP genotype including alanine136 and glutamine171. [file 12711_2021_646_MOESM2_ESM.docx]

**Additional file 2.**

**Table S2.** Product-limit estimates of median survival (days) for naturally scrapie-infected sheep and goats homozygous for the wild type *PRNP* genotype including alanine_136_ and glutamine_171_.

Groups of naturally infected research animals were born and raised at the Animal Disease Research Unit between 2013 and 2015. Natural exposure included being raised by a scrapie-infected dam and living within a dry lot environment amongst other scrapie-infected animals of the same species. All males were wethers. Animals were culled as soon as progressive signs consistent with clinical scrapie disease were noted. These animals (or their dams) were negative for detection of SRLV by annual serology. The PROC LIFETEST procedure (SAS 9.4) was used to conduct a homogeneity test of strata (goats, sheep) while controlling for gender.

|  | **Female** | |  | **Male** | |  | | **All** | |  |
| --- | --- | --- | --- | --- | --- | --- | --- | --- | --- | --- |
|  | **N** | **Median (95%CL)** |  | **N** | **Median (95%CL)** | |  | | **Median (95%CL)** | |
| **Goat** | 10 | 1220 (969-1284) |  | 6 | 1296 (1134-1681) | |  | | 1221.5 (1134-1341) | |
| **Sheep** | 9 | 1065 (775-1115) |  | 11 | 1030 (743-1079) | |  | | 1039.5 (855-1079) | |
